# The Reality of Uncertainty in Mental Health Care Settings Seeking Professional Integration: A Mixed-Methods Approach

**DOI:** 10.5334/ijic.4168

**Published:** 2018-12-19

**Authors:** Chiara Pomare, Louise A. Ellis, Kate Churruca, Janet C. Long, Jeffrey Braithwaite

**Affiliations:** 1 Centre for Healthcare Resilience and Implementation Science, Australian Institute of Health Innovation, Macquarie University, AU

**Keywords:** integrated care, professional integration, mental health, uncertainty, complexity, mixed-methods

## Abstract

**Introduction:**

Uncertainty is a common experience in the complex adaptive health system, particularly amongst mental health professionals structured for the delivery of integrated care. Increased understanding of uncertainty will not necessarily make things more certain, but can act to sensitize professionals to the challenges they face. The aim of this study is to examine the types and situations of uncertainty experienced by professionals working in a mental health setting based on an integrated care model. The research assesses the impact of experience and professional group on reported uncertainties.

**Methods:**

First, semi-structured interviews were undertaken with clinical and non-clinical staff to examine uncertainties experienced by professionals working in headspace centres in Australia. Second, an online survey was conducted to quantify the experiences of uncertainty and explore associations.

**Results:**

Findings revealed three overarching and largely interrelated aspects of uncertainty, namely: decision-making; professional role; and external factors. Most commonly, staff reported experiences of uncertainty pertaining to deciding to accept a client into the service and then deciding how to treat them. This is often due to arbitrary, or overly-restrictive criteria in integrated care. Findings also suggested that uncertainty does not necessarily decline with experience and there were no significant differences in levels of uncertainty between clinical and non-clinical staff.

**Conclusions:**

This study highlights the importance of acknowledging uncertainties and actively clarifying role ambiguities when working alongside diverse professionals in mental health care.

## Introduction

The rapid growth of complexity in health care [1], and specifically, in care settings where team-oriented, integrated care is pursued [2,3], has brought with it increased uncertainty [4]. Uncertainty in health care emerges in situations infused with fluctuating probability, high ambiguity, and intricate complexity [5]. Situations of uncertainty include: deciding on a diagnosis when signs and symptoms are unclear [5]; trying to apply abstract criteria to real-world problems [6]; or facing the unknowable predictability of disease trajectory [7]. Uncertainty, in its many guises, is all-too-often “uncomfortable” [8 p. 104], and attempts by health care professionals to tackle it may lead to a sense of “personal inadequacy” [9, p. 210]. Some suggest that rather than attempt to overcome uncertainty, the first step is to recognise and acknowledge its presence [10]. This begins with attempting to understand uncertainty as it is experienced by those on the frontlines of complex, team-oriented, integrated models of care.

Understanding, characterising, and assessing uncertainty in health care is an important area of research. This is because uncertainty is a frequent experience in health care [5], and may give rise to disempowering feelings [11] and accountability issues [12] that can disrupt the efficacy of care delivery. For example, a study of nurses in a changing health system showed that uncertainty related to job security was associated with poor physical health and high levels of depression for nursing staff [13]. A qualitative study of medical residents’ experiences in clinical decision making revealed that uncertainty can result in compromised patient care [14]. All-in-all, uncertainty is a common experience in health care that can negatively affect staff and patients.

One strand of research within the uncertainty literature examines the role of experience. For some, uncertainty stems from inexperience [15] or deficits in knowledge [16], otherwise termed ‘technical uncertainty’ [6]. That is, uncertainty in health care is simply a result of not knowing or not having experienced the event or circumstances in question. A contrasting and more recent view argues that uncertainty is less about what can be learned or gained from experience, and more related to what is unknowable [5]. According to Han et al. [5] uncertainty across various aspects of care is riddled with properties such as contingencies, equivocacy, and intricacies that cannot be overcome by levels of experience or increased knowledge. For example, contingencies may cloud the decision-making processes of health care staff; intricacies in navigating roles and responsibilities may make it difficult to proceed on an issue; equivocacy over the future effect of a particular medication may lead to uncertainty for doctors’ prescribing decisions. However, there is a gap in the literature in exploring if experience mitigates issues of professional uncertainty in health care.

Research assessing experiences of uncertainty in health care has largely focused on the practices or perspectives of clinical staff [e.g. 6,14,17], taking little consideration of other actors in the health care system, such as non-clinical staff. Non-clinical staff are often the first point of contact for a patient and must juggle diverse roles and responsibilities. Thus, it is not surprising that these staff members experience job demand stressors that lead to emotional exhaustion and issues of role ambiguity [18]. However, the nature of these uncertainties beyond role ambiguity has not yet been examined.

There have been several reviews in the health care literature attempting to classify experiences of uncertainty [5,10,17]. However, much of this research has lacked a specific focus on the uncertainties faced in the domain of mental health care, a sector in the health care system often excluded from research endeavours [19]. Uncertainty is of particular concern in mental health care because diagnoses of psychological disorders are often overlapping and criteria frequently change [20]. Unlike other domains of health care, there is no equivalent to, for example, a blood test, to diagnose a mental health condition [21]. Past research exploring uncertainty in mental health care has shown that even when a clinical decision is reached, there remains uncertainty in the behavioural outcome of the client [22] and the outcome of the condition itself [7]. For example, professionals cannot readily predict violence of some mental health care patients [22]. Adding to this complexity, mental health care delivery has been increasingly re-conceptualised into models of integrated care, involving the co-location and interdisciplinary working of various health care professionals, from mental health, physical health, and social care [23]; otherwise termed professional integration [24]. While beneficial in improving health outcomes in comparison to usual models of care [25], integrated care models (albeit only on the meso-level of professional integration) may lead to new problems such as sharing responsibility of patients, which may bring about uncertainties regarding indistinct role boundaries [2].

While models of professional integration in mental health care have been taken up internationally [23], there is considerable space in the literature to examine the experiences of professionals working within this domain. Aside from recognising the complexity of such a service configuration [2,3], and therefore the propensity for unpredictability and ambiguity, we know little about additional sources and types of uncertainty faced by professionals working in mental health care settings structured along the lines of professional integration. To tackle this research deficit, this study aimed to examine the types and situations of uncertainty experienced by professionals, both clinical and non-clinical, in mental health care (using *headspace* Australia, a mental health care arrangement modelled on professional integration, as a case exemplar). Based on past research, we hypothesised that non-clinical staff will report similar experiences of uncertainty to clinical staff, largely surrounding issues of role and unclear diagnostic and treatment decisions. In support of the definition of uncertainty as an unknowable phenomenon [5], we also hypothesised that professional experience would have no significant impact on reported uncertainties.

## Methods

We applied a sequential, mixed-methods approach, collecting qualitative data through semi-structured interviews, which then informed the construction of an online survey that provided quantitative attitudinal data. The study received ethical approval from the Macquarie University, Sydney, Human Research Ethics Committee [HREC ref 5201700297] and local sites granted approval for site access and data collection, with individual participants providing consent to participate. The study ran through calendar year 2017.

### Study Setting and Participants

Participants were professionals (clinical and non-clinical) working in mental health care facilities: two *headspace* centres located in metropolitan Australia. *Headspace* is Australia’s National Youth Mental Health Foundation that aims to facilitate and promote improvement in youth mental health, social wellbeing and economic participation of young Australians between 12–25 years of age [26]. It is a government-funded initiative with over 100 centres located Australia-wide, which have been set up by the overarching body (head office).

According to the Rainbow Model of Integrated Care, *headspace* is a model of professional integration, whereby the facility strives for interprofessional partnerships based on shared accountability and responsibility to deliver care [24]. That is, various health care professionals: general practitioners, psychologists, psychiatrists, and other health professionals are collectively responsible for providing care to a defined population of young Australians. Professionals not only work together side by side, but collectively make decisions in daily team meetings, share medical records, and are collectively responsible for client cohorts.

Staff employed at these *headspace* centres are part- or full-time clinicians, or managerial and administrative staff, such as practice managers. The facility is community based, allowing for walk in and appointment clients, and refers to other services if the client does not meet the *headspace* criteria (i.e., younger than 25 years and considered ‘early intervention’). The two *headspace* centres that participated in this study were similar in their physical layout, operational structure, and number and qualification of employees. One had been opened recently, with the other in operation for over a decade.

### Semi-Structured Interviews

Semi-structured interviews were conducted to explore and identify uncertainties faced by professionals in the integrated mental health model. Seven participants, from both centres, participated in the semi-structured interviews. Participants were chosen via purposive sampling [27] with the intention to capture a mix of clinical and non-clinical employees of varied experience who could provide a breadth of insight into uncertainties encountered by staff. While all staff were invited, sampling was chosen based on suggestions made by managerial staff. Interviews took place at the participants’ place of work and informed consent was provided before the commencement of the interviews.

Questions were open-ended and developed in consultation with experts in the field to ensure applicability to the various roles of professionals working in mental health care. The interview schedule included questions about professional uncertainty in mental health care: what it is, who experiences it, and specific examples (Appendix A). Interviews were audio recorded and professionally transcribed. Transcripts were then imported into NVivo software, Version 11.4, for coding and qualitative data analysis. Qualitative analysis involved thematic analysis [28] using an open coding process [29] to identify the types of uncertainty. The analysis was based on inductive coding of content and deductive work by considering theoretical concepts identified in the literature [5,6]. Interviews were led by the first author (CP) and data analysis was conducted by two researchers (CP and KC) to triangulate findings, ensuring their credibility [30].

### Online Survey

The online survey (Appendix B) was designed as a result of the interview findings, to confirm and quantify the manifestations of the “types” and “situations” of uncertainty revealed in the qualitative analysis. All staff at the two *headspace* centres were invited to participate, giving a study population of 50 potential participants. The survey was piloted (*N* = 10) with a sample of experts in the field outside of the research sites before administering it to them. Subjects were invited to participate in the survey by an email sent to their work email address by the research team via their manager, to help increase response rates. The survey contained a secure URL link. The link directed them to a secure online survey platform, Qualtrics, hosted by Macquarie University. One follow-up reminder email was sent to staff two weeks after first contact.

The survey began with an information and consent form, followed by a demographic questionnaire and enquiry into experiences of professional uncertainty. Participants were asked to indicate the prevalence of a number of situations of uncertainty at their workplace in the past 6 months: “Below is a list of situations of professional uncertainty. Please indicate if YOU have experienced uncertainty in any of these situation(s) in the LAST 6 MONTHS. Please also consider if you have observed or been aware of OTHER staff of *headspace* being uncertain about the following situation(s)”. An open-ended question asked participants to disclose any additional situations of uncertainty not listed. The survey was estimated to take approximately 5–10 minutes to complete. Statistical analysis of survey responses, including descriptive and comparison analyses, was conducted using the software program SPSS Version 23.0.

## Results

### Qualitative: Interviews

Seven participants took part in the semi-structured interviews; five were female and two were male; five were in clinical positions, and two in non-clinical (management) roles; four were in senior roles, with three in junior positions. Interviews averaged approximately 40 minutes. Thematic analysis led to the identification of three types of uncertainty (deciding next steps; professional role; and external factors) and six more specific situations of uncertainty (system services and processes; unpredictability of client outcomes; are we the best service?; what option should I chose?; am I doing this right?; and demarcation of roles). Uncertainties were hierarchically classified, whereby each type of uncertainty encapsulated two specific situations. Table 1 provides explanations and examples of the “types” and “situations” of uncertainty.

**Table 1:** Types and situations of uncertainty.

Type	Situation	Explanation	Exemplar Data

Deciding Next Steps	Are we the best service?	Deciding if the client meets inclusion criteria for the service.	“are we the best service to assist this young person?” (NC1)
	What option should I chose?	There are many possible trajectories to take in terms of treatment options and follow-up, professionals must decide on the best suited path for the client.	“it’s quite tricky. Unless you fit into some certain boxes it’s quite challenging” (NC1)
Professional Role	Am I doing this right?	Self-doubt in professional competency.	“Addressing the young person’s presenting issue while also making sure that you don’t leave all the other stuff that they’ve got going on” (C3)
	Demarcation of roles	Lack of clarity between or within roles.	“things do get blurred, where a lot of the time I do find myself doing things that I’m like well this is really not my job” (C2)
External Factors	System services & processes	The sustainability of the service and projects.	“well at this stage we’re funded till [date removed]. But we don’t know what it’s going to look like past that date” (NC1)
	Unpredictability of client outcomes	Unforeseen and uncontrollable client outcomes and behaviours.	“because you can’t tell, with certain clients it might end up being nothing an hour later and it’s fine and we’ll go about our whole day whereas it could end up being a potentially fatal situation” (C2)

An additional major theme identified in the interview data involved the interrelated nature of the uncertainties experienced by professionals working in interprofessional relationships in this mental health care setting. That is, an uncertain situation rarely occurred in isolation from others; it might feed into or drive other uncertainties. For example, one participant discussed the uncertainty experienced regarding the outcome of a client; this ambiguity about the future created uncertainties in deciding the next steps and also led to a questioning of professional role:

“if there’s suicidal intent, and plans, I guess there can be some uncertainty in there – is this person safe to go home? Is this person safe to leave here with someone else? Do they need to go to hospital?” (C4).

Hence participants’ accounts suggested it was rare to experience only one situation of professional uncertainty due to contingencies and interactions among unknown issues and events.

### Quantitative: Survey

**Figure 1:**
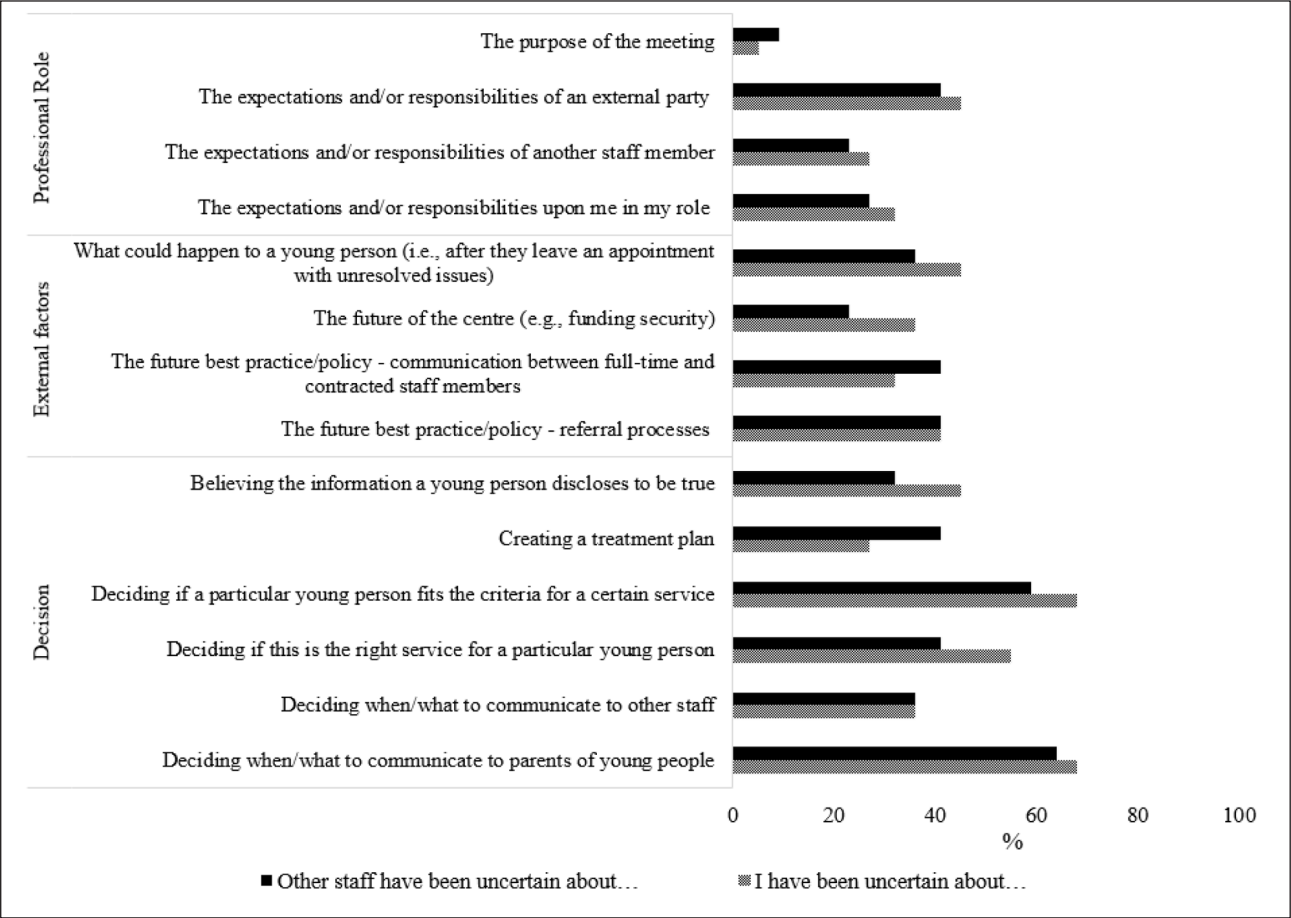
Reported experiences of uncertainty.

Almost half (*N* = 24, 48%) of the 50 potential respondents invited to participate, completed the online survey. Participants’ demographic information is presented in Table 2. Average scores were computed to determine the most frequently reported type of uncertainty. On average, decision uncertainty (deciding next steps) was the most frequently reported uncertainty by participants (*M* = 0.47, *SD* = 0.30), followed by external factors uncertainty (*M* = 0.34, *SD* = 0.30) and professional role uncertainty (*M* = 0.25, *SD* = 0.28) (See Figure 1). Responses to open ended questions asking for any other scenarios of uncertainty were consistent with situations already identified in the qualitative component of the research.

**Table 2:** Staff characteristics of questionnaire respondents.

Demographic Details		

Gender	Male	6
	Female	18
Contract of Employment	Full time	11
	Part time	13
Duration of Employment at Current Place of Work	<6 months	7
	6 months–2 years	10
	2+ years	7
Professional Qualification	Psychiatry	2
	Psychology	7
	Other medicine	2
	Administration	6
	Other*	7

*Counselling, Nursing, Social Work, Occupation Therapy.

Professional groups were classified into clinical and non-clinical groups (i.e., management and administrative staff), in order to assess the prevalence of uncertainties experienced by different professional roles in the centres. Mean scores were computed to indicate the total professional uncertainties experienced for each overarching type of uncertainty. In support of the hypothesis, independent sample t-tests revealed no significant differences in the uncertainties reported between groups of clinicians and non-clinicians for decision uncertainties (*t*(22) = 0.80, *p* = .43), role uncertainties (*t*(22) = 0.08, *p* = .70), and uncertainties pertaining to external factors (*t*(22) = –1.89, *p* = .07). Testing revealed a significant difference in the scores for full-time (*M* = 0.50, *SD* = 0.27) and part-time staff (*M* = 0.21, *SD* = 0.27): *t*(22) = 2.61, *p* = .02. This indicates that full time staff are more likely to experience uncertainty related to external factors (such as uncertainty about funding and job security), compared to staff that are only there on a part time basis. This significance was not found for the other types of decision (*t*(22) = 0.07, *p* = .95) and role uncertainty (*t*(22) = 1.12, *p* = .28).

Despite a difference in the longevity of operation of the two *headspace* centres, no significant differences were found between the two centres for decision uncertainties (*t*(22) = –0.75, *p* = .47), role uncertainties (*t*(22) = –1.12, *p* = .28), and uncertainties pertaining to external factors (*t*(22) = 0.04, *p* = .97). Experience was assessed using Pearson correlational analyses to examine the association between years of experience working at *headspace* and situations of uncertainty; correlational analyses did not reach significance for any situation of uncertainty, supporting the hypothesis that uncertainties are not dependent upon professional experience.

## Discussion

This study offers insights into the reality of uncertainty for professionals working in two sites organised for professionally integrated mental health care (*headspace* centres in Australia). Thematic analysis of semi-structured interviews revealed three prominent types, and six more specific situations, of uncertainty. Subsequent quantitative analysis of survey responses demonstrated that these types and situations are commonly experienced by a large proportion of staff working in *headspace* centres, including primary care physicians, mental health care specialists, general intake clinicians, and administrative and management staff. No significant differences were found between clinical and non-clinical staff. For the two facilities, one which had opened recently and the other with a decade operating, there were no significant differences in the uncertainties reported between the two *headspace* centres, nor were significant associations found between the level of an individual’s professional experience and the degree to which he or she experienced situations of uncertainty. However, modest differences were observed dependent upon the contract of employment, with part-time staff experiencing less uncertainty than full-time staff. This is suggestive of the proposition that the more you have only one source of income (full timers), the more you worry about losing it. We explore the findings and implications of this research in more detail below.

### Decision-Making Uncertainty

Decisional uncertainty is regarded as a common experience in health care [31]. Past research has provided examples, for instance, that nurses experienced uncertainty when deciding whether or not the patient meets criteria to call the medical emergency team [32], and clinicians report uncertainty when deciding on a child’s quality of life in a neonatal ward [31]. In mental health care, past research has found that general practitioners experienced uncertainty when deciding on diagnoses of mental health conditions [33]. For the present study, decisional uncertainty was the most commonly reported type of uncertainty among professionals working in *headspace* centres. Experiences of decision-making uncertainty were categorised into two, seemingly sequential, decisions: “does the presenting client meet the inclusion criteria of the service?” and, then, “what to do when the client is in the service?” For the participants, these uncertainties were rooted in the ambiguous criteria of the service within which they worked: *headspace*, a mental health care model of professional integration in Australia. The *headspace* model is restricted to youth, and focuses on early intervention [26], however, these results indicate that who qualifies as ‘early intervention’ is often unclear. Interestingly, this study identified this as a challenge for non-clinical as well as clinical staff, perhaps because non-clinical staff often manage enquiries made to the service by young people. The present study revealed no significant association between professional experience and experiences of uncertainty, indicating that such examples of decision-making in mental health care may never become clear. This is because the source of such a decision is grounded in ambiguity, probability, and complexity [5]; thus, the “correct” decision may be perceived as unknowable, rather than unknown.

### Professional Role Uncertainty

Uncertainty pertaining to professional role involves any situation of uncertainty related to one’s job, tasks or work performance. In mental health care settings pursuing professional integration, various professionals of different expertise (psychologist, psychiatrist, general practitioner, nurse, counsellor etc.) work in an interdisciplinary way, sharing responsibility for clients and making collective decisions. The nature of professional integration may lead to blurring of boundaries and subsequent experiences of uncertainty. Uncertainty regarding role (i.e., role conflict or role ambiguity) is a common stressor among mental health care workers [34], and has also been reported more broadly in the health care literature. For example, absence of clarity between the roles of doctors and nurses has been reported for more than a decade [12,35], and clinicians have reported “not knowing how to act” when dealing with patients of different ethnicities [11], or feeling uncertain about responsibilities for a patient after patient transfer [36]. In the present study, these uncertainties of role persisted for participants with over five years’ experience working at *headspace*, highlighting the pervasive nature of these uncertain situations and suggesting that staff do not become better at resolving or reducing role uncertainty based on greater experience.

### External Factors Uncertainty

Participants also reported uncertainty related to external factors, specifically: unpredictability of client outcomes, and unpredictability of system services and processes. These uncertainties were thematically categorised as external because participants typically had limited capacity to control them (e.g. you can’t control what a client will do with the advice you give them nor the prognosis of that client). They were not associated with professional group or experience. Unpredictability of client outcomes is a situation of uncertainty not specific to mental health care, and has also been observed, more broadly, in other domains of health care. For example, the uncertainty of the illness trajectory in palliative care [37] is consistent with the uncertain illness trajectory and behavioural outcomes reported in the present study, and previous research into mental health care [22]. However, the second situation of external uncertainty—unpredictability of system services and process—may be specific to *headspace* centres. Unlike public hospitals that are, for the most part, confident in the longevity of their organisation, uncertainty of the sustainability of the service may be more an issue for community-based health care organisations that rely on relatively new funding sources, such as *headspace*. Results showed that this is a particular uncertain situation experienced more by full-time staff, compared to part-time staff, which is understandable given the greater impact it would have on their stability of employment and potential future income levels.

### Implications and Recommendations

The present study serves as a pivot point for acknowledging the veracity of uncertainty in *headspace* centres and exploring attempts to manage it. Looking beyond *headspace*, there is a growing international trend for models of youth health care aiming for professional integration; thus these issues of uncertainty may be applicable elsewhere in similar settings [23]. In acknowledging the various types and situations of uncertainty as presented in this study (decision, role, external factors), we suggest that how we manage uncertainty should also vary by situation. The present study indicated that having years of professional experience is not enough to avoid uncertainty; this means that despite how long you work in *headspace* centres, some aspects will remain ‘unknowable’. This is particularly the case in situations of decision uncertainty and external factors uncertainty, where the correct decision or future behaviour of a client can never be known with complete clarity. Despite claims in the literature that team meetings provide optimal opportunity for professionals with an array of expertise to discuss complex cases and tackle uncertainties [38], the present findings suggested that this may not be the sole solution. Our recommendations for managing uncertainty are two-fold: (1) Professionals working together in mental health care settings should make conversations of uncertainty the norm. This can be achieved by formally acknowledging uncertain experiences of staff. (2) Managers and senior staff should seek to actively promote conversations of decision and external factors uncertainty, without necessarily attempting to find a solution.

This recommendation does not apply to all types of uncertainty. Role ambiguity, unlike decision and external factors uncertainty can, to some extent, be clarified. The present study highlighted that uncertainty in your role was not determined by the experience you have. We argue that rather than experience, active clarification is needed. That is, while a professional may have over five years’ experience working at *headspace* (or other settings organised for professional integration), the ambiguity of roles and responsibilities may never be clarified without an explicit discussion. These uncertainties need to be addressed formally and include ancillary (non-clinical) staff. This can be done through the development of organisational documents from head office and negotiated locally between staff. Therefore, people in complex care settings should focus on addressing the uncertainties that can be resolved (role ambiguity) by clarification and demarcation of blurred roles and responsibilities, while continuing to acknowledge and normalise conversations of ‘unknowable’ uncertainty (decision and external factors).

### Strengths and Limitations

The strengths of this study lie in its sequential, mixed-methods design that allowed for the exploration and analysis of a previously under-researched topic. The application of triangulation processes is designed to create levels of reliability and validity; multiple coders and the use of mixed-methods, improve the rigour in the findings and can act to negate common criticisms of subjectivity and bias of qualitative research [30]. Limitations of this study lie in its potential for bias. Sampling of interview participants was conducted with assistance by managerial staff, and staff were sent an email to participate in the survey via centre management, at *headspace’s* request, in an attempt to increase participation rates. To mitigate bias, all efforts were made to capture a diverse sample (see Table 2) and, for the survey, participants were informed that their responses would remain anonymous. Further, while semi-structured interviews were only conducted with seven participants, they were reflective of the population (50 staff members). Despite the small sample size, discussions were in-depth and met the saturation criterion. We note, however, that additional forms of qualitative enquiry, such as focus groups, may have provided more in-depth perspectives and potential solutions to issues of uncertainty faced by *headspace* staff; this is an avenue for future research. Further, while the validity of this study may be restricted by the survey response rate (48%), we sought to attain rigour from statistical analysis; and triangulation was sought through the integration of combined qualitative and quantitative data.

## Conclusion

This research explored uncertainties experienced by clinical and non-clinical staff navigating the complex system of mental health care, using two Australian *headspace* centres aligning for professional integration as research sites. Findings revealed that clinical and non-clinical staff experience uncertainty related to: decision-making, professional role, and external factors. Uncertain situations were interrelated and did not appear to diminish with increased experience. The types of uncertainty reported in this study are generally consistent with the health care literature, suggesting that uncertainties experienced by professionals working in integrated settings of mental health care may not be so different to uncertainties experienced in other domains of care. We recommend formal acknowledgement of the veracity of uncertainty and attempts to normalise conversations about uncertainty. Further, in mental health care settings pursuing professional integration, facilities should focus on clarifying role ambiguities that appear to be resistant to experience.

## Additional Files

The additional files for this article can be found as follows:

10.5334/ijic.4168.s1 Appendix A. Semi-Structured Interview Schedule.

10.5334/ijic.4168.s2 Appendix B. Survey.
