# Supplementary material for: The Reality of Uncertainty in Mental Health Care Settings Seeking Professional Integration: A Mixed-Methods Approach
Source: Int J Integr Care. 2018 Dec 19;18(4):13. doi: 10.5334/ijic.4168 (PMC6300768; doi:10.5334/ijic.4168)
Supplement: Appendix A. — Semi-Structured Interview Schedule. [file ijic-18-4-4168-s1.pdf]

(6). Appendix A

(6.1). Semi-Structured Interview Schedule

Topics	Questions
Role	• Would you mind telling me about your role at *headspace?*<br>• What sort of interactions do you have with the *headspace* clients?<br>• What sort of interactions do you have with your *headspace* colleagues?
Definition & understanding	*In today's interview, we are going to talk largely about uncertainty. In health care, uncertainty is an inevitable phenomenon associated with ambiguity, probability and complexity. In simple terms, uncertainty is the state of being unsure.*<br>• In your own words, what does professional uncertainty mean specifically for professionals working in mental health care?
General experiences	• Is there a particular scenario of professional uncertainty that comes to mind?
Specific events	*Now we are going to talk about particular events of professional uncertainty in mental health care; we will talk about six specific topics.*<br>• Do you think * is an area of professional uncertainty in mental health care?<br>*(Topics included*: diagnosis; prognosis; causal explanation; treatment recommendations; health care systems; communication.)*
Closing questions	• Out of all these uncertainties we have discussed today, in your opinion, what is the most prominent?<br>• In your opinion, how does *headspace* deal with situations of professional uncertainty?
