# Supplementary material for: The Reality of Uncertainty in Mental Health Care Settings Seeking Professional Integration: A Mixed-Methods Approach
Source: Int J Integr Care. 2018 Dec 19;18(4):13. doi: 10.5334/ijic.4168 (PMC6300768; doi:10.5334/ijic.4168)
Supplement: Appendix B. — Survey. [file ijic-18-4-4168-s2.pdf]

(7). Appendix B

(7.1). Survey

Welcome!

You are invited to participate in a study on professional uncertainty in mental health care. You have been chosen to participate in this survey because you are a staff member of headspace - our aim is for ALL staff at your centre to complete this survey! It will take around 10 minutes to complete.

Uncertain about discussing uncertainty?

Uncertainty is commonly described in the literature as a normative experience among health care professionals – acknowledging instances of uncertainty is not a weakness but an integral part of working in health care, particularly mental health care. You will remain anonymous and unidentifiable in any write up of the results.

About me!

1. Gender:
    - o Male
    - o Female
    - o Other
2. How long have you worked at headspace?
    - o < 3 months
    - o 3 - 6 months
    - o 6 months – 1 year
    - o 1 – 2 years
    - o 2 – 5 years
    - o 5+ years
3. In terms of your role at headspace, which of the following professional groups would you <u>most closely</u> identify with? (Please select one)
    - o Youth Access team
    - o Management/leadership team
    - o Contracted clinicians
    - o Administration
    - o Other (please specify: ……………………………………)
4. What is the field of your professional qualification?
    - o Psychiatry
    - o Psychology
    - o Counselling
    - o Medicine (GP)
    - o Nursing
    - o Social Work
    - o Occupational Therapy
    - o Admin

- Other (please specify: ……………………………………)

5. Which of the following best describes your role?
   - Full time
   - Part time
   - Casual
   - Other (please specify: ……………………………………)
6. Which of the following best describes you?
   - Senior staff member
   - Junior staff member
   - Not sure

Situations of professional uncertainty

Below is a list of situations of professional uncertainty. Please indicate if YOU have experienced uncertainty in any of these situation(s) in the last 6 months. Please also consider if you have observed or been aware of OTHER staff of headspace being uncertain about the following situation(s).

1. Please select all that apply for both columns.

	I am/have been uncertain about…	*Other staff at headspace have been uncertain about…*
Deciding when/what to communicate to **parents of young people**	○	○
Deciding when/what to communicate to **other staff**	○	○
Deciding if headspace is the right service for a particular young person	○	○
Deciding if a particular young person fits the criteria for a certain service	○	○
Creating a treatment plan	○	○
Believing the information a young person discloses to be true	○	○
The best practice/policy that headspace should follow in terms of **referral processes**	○	○
The best practice/policy that headspace should follow in terms of **communication between full-time and contracted staff members**	○	○
The future of headspace (e.g., funding security)	○	○
What could happen to a young person (i.e., after they leave an appointment with unresolved issues)	○	○
The expectations and/or responsibilities upon me in **my role**	○	○

(or **their** role, respectively)		
The expectations and/or responsibilities of **another staff members**	○	○
The expectations and/or responsibilities of an **external party** (i.e., a service that you have had contact with but is not part of headspace)	○	○
The purpose of the meeting	○	○
None of the above		

2. Are there any other situations of uncertainty experienced by headspace staff you are aware of?
